# Supplementary material for: Exploring the Pathological Effect of Aβ42 Oligomers on Neural Networks in Primary Cortical Neuron Culture
Source: Int J Mol Sci. 2023 Apr 2;24(7):6641. doi: 10.3390/ijms24076641 (PMC10094920; doi:10.3390/ijms24076641)
Supplement: Supplementary file 1 [file ijms-24-06641-s001.zip › ijms-2316749-supplementary.pdf]

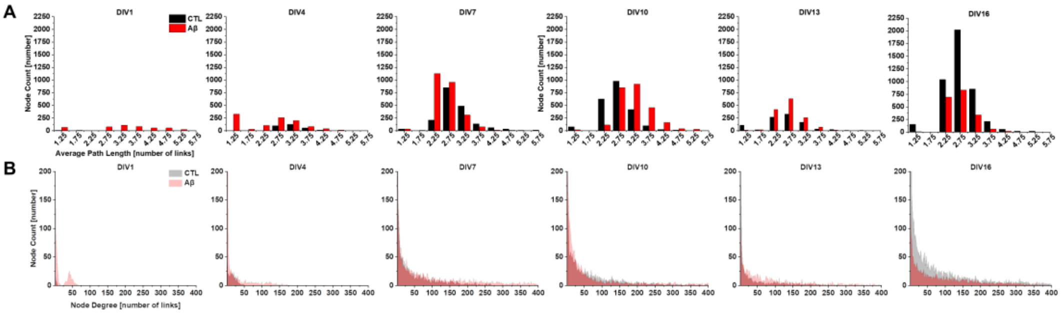


**Supplementary Figure S1. (A) Distribution histogram of average path length in number of links to node count. Control (black) and Aβ42 oligomer treatment (red) groups in DIV 1, 4, 7, 10, 13 and 16. (B) Distribution histogram of node degree in number of links to node count.**

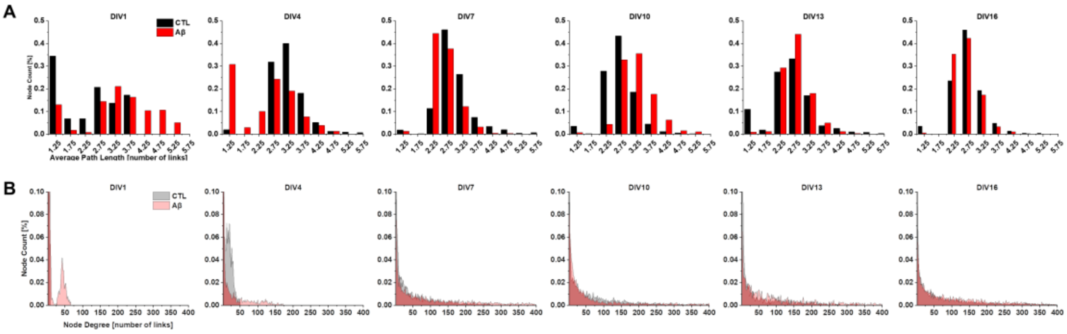


**Supplementary Figure S2. (A) Distribution histogram of probabilistic path length in number of links to node count percentage. Control (black) and Aβ42 oligomer treatment (red) groups in DIV 1, 4, 7, 10, 13 and 16. (B) Distribution histogram of probabilistic node degree in number of links to node count percentage.**

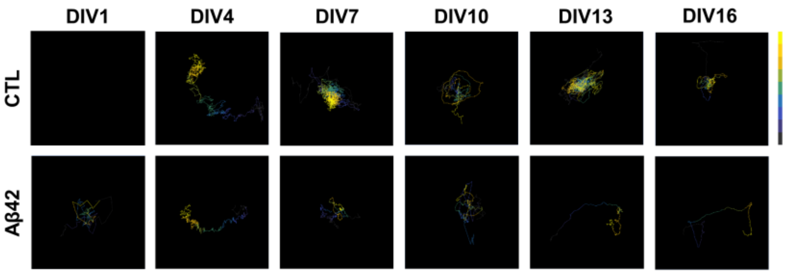


**Supplementary Figure S3. (A) Single representative CAT from the intermingled trajectories in figure 4. Control (CTL) and Aβ42 oligomer treatment groups in DIV 1, 4, 7, 10, 13 and 16. The color scale bar represents an initiation (gray) to termination (yellow).**
